# Cranberry Extract Standardized for Proanthocyanidins Promotes the Immune Response of *Caenorhabditis elegans* to *Vibrio cholerae* through the p38 MAPK Pathway and HSF-1

**DOI:** 10.1371/journal.pone.0103290

**Published:** 2014-07-25

**Authors:** Jessica Dinh, Joseph T. Angeloni, Daniel B. Pederson, Xiaoxia Wang, Min Cao, Yuqing Dong

**Affiliations:** 1 Department of Biological Sciences, Clemson University, Clemson, South Carolina, United States of America; 2 Institute for Engaged Aging, Clemson University, Clemson, South Carolina, United States of America; Duke University Medical Center, United States of America

## Abstract

Botanicals are rich in bioactive compounds, and some offer numerous beneficial effects to animal and human health when consumed. It is well known that phytochemicals in cranberries have anti-oxidative and antimicrobial activities. Recently, an increasing body of evidence has demonstrated that cranberry phytochemicals may have potential benefits that promote healthy aging. Here, we use *Caenorhabditis elegans* as a model to show that water-soluble cranberry extract standardized to 4.0% proanthocyanidins (WCESP), a major component of cranberries, can enhance host innate immunity to resist against *Vibrio cholerae* (*V. cholerae*; wild type C6706 (O1 El Tor biotype)) infection. Supplementation of WCESP did not significantly alter the intestinal colonization of *V. cholerae*, but upregulated the expression of *C. elegans* innate immune genes, such as *clec-46*, *clec-71*, *fmo-2*, *pqn-5* and C23G10.1. Additionally, WCESP treatment did not affect the growth of *V. cholerae* and expression of the major bacterial virulence genes, and only slightly reduced bacterial colonization within *C. elegans* intestine. These findings indicate that the major components of WCESP, including proanthocyanidins (PACs), may play an important role in enhancing the host innate immunity. Moreover, we engaged *C. elegans* mutants and identified that the p38 MAPK signaling, insulin/IGF-1 signaling (IIS), and HSF-1 play pivotal roles in the WCESP-mediated host immune response. Considering the level of conservation between the innate immune pathways of *C. elegans* and humans, the results of this study suggest that WCESP may also play an immunity-promoting role in higher order organisms.

## Introduction

A broad array of evidence demonstrates a positive link between plant phytochemicals and health-promoting effects, such as cancer prevention, bone health, a reduced risk of cardiovascular and metabolic diseases, etc. [1]–[12]. Thus, many edible fruits, including cranberries, have caught people's attention. American cranberry (*Vaccinium macrocarpon*) is rich in various polyphenols, including anthocyanins, flavonols, proanthocyanidins (PACs), phenolic acids, stilbenes, etc. [13], which have been implicated in numerous health benefits. For instance, cranberries' A-type PACs may prevent urinary tract infections (UTI) by inhibiting the adhesion of P-fimbriated *Escherichia coli* to uroepithelial cells [14], [15]. Flavonols, salicylic acid, and resveratrol derived from cranberries showed beneficial activity against inflammation in animal models [3], [13]. Furthermore, ingestion of cranberry extract rich in flavonoids decreased the growth rate and size of tumors in mice models [16]. Recently, studies in model systems demonstrated that the water-soluble cranberry extract standardized to 4.0% PACs (WCESP) can promote animals' response to abiotic stress accompanied with a prolonged lifespan [17]–[21]. In this context, it is of interest to examine whether this WCESP may also promote the host response to biotic stressors, such as pathogenic bacteria.

The nematode *Caenorhabditis elegans* (*C. elegans*) has been engaged as an infection model to study host-pathogen interactions. Since *C. elegans* can be easily exposed to a variety of pathogens without ethical issues, this model has been successfully used to identify and assess virulence factors of several human pathogens, e.g., *Pseudomonas aeruginosa, Yersinia pseudotuberculosis, Salmonella enterica, and Vibrio cholerae* [22]–[26]. Additionally, *C. elegans* also makes a good model for studying host innate immunity from an evolutionary perspective [27], [28]. As a predator of bacteria, *C. elegans* has evolved a complex innate immunity to survive in the natural environment. Their immune responses include physical barriers, avoidance behaviors, secretion of antimicrobial molecules, etc. [27], and these defenses are regulated by a number of signaling pathways. Of note, the most significant defense regulatory signaling pathways in *C. elegans* are evolutionarily conserved, such as the p38 mitogen-activated protein kinase (MAPK) pathway, the insulin/insulin-like growth factor-1 signaling (IIS) pathway, the transforming growth factor β (TGF-β) signaling pathway [29]–[32]. Furthermore, the phenotypic changes normally associated with bacterial infections, like animal mortality, motility and fecundity, can be examined noninvasively in this model.

Cholera, mediated by *Vibrio cholerae* (*V. cholerae*), is a severe public and individual health problem around the world. To understand the biology of this disease, it is equally critical to 1) identify and understand the major virulence factors of *V. cholerae* and 2) understand the host gene expression response to *V. cholerae* infection. Although many virulence factors of *V. cholerae*, such as cholera toxin (CT), toxin-coregulated pili (TCP), and hemolysin, have been identified and researched extensively, questions regarding the host response to *V. cholerae* infection still remain unanswered [33], [34]. To answer these questions, *C. elegans* has been introduced to study the interactions with V. cholerae by several research groups. Previous studies showed that *V. cholerae* can cause lethal infection in *C. elegans* and this lethality was associated with the bacterial hemolysin HlyA and the extracellular protease PrtV, which are regulated by the quorum sensing LuxO-HapR regulatory pathway [26], [35]. Genomic studies of the *C. elegans* host model have identified many innate immune genes which are induced upon *V. cholerae* infection [36]. Based on this knowledge, we sought to establish the *C. elegans* infection model as a powerful platform to investigate the protection mechanisms of nutraceuticals and pharmaceuticals against *V. cholerae* and other bacterial pathogens.

In the present study, we attempted to examine the potential effects of WCESP on host immune response to *V. cholerae* and its underlying mechanisms in *C. elegans*. We found that supplementation of WCESP prolonged the survival time of worms exposed to V. cholerae, implying the protective effects of WCESP against V. cholerae. Utilizing worm mutants, our genetic epistasis analyses suggested that WCESP may act through the p38 MAPK signaling pathway, IIS and HSF-1, but independent of DAF-16, to prevent killing by V. cholerae. Notably, WCESP administration significantly modulated the expression of host innate immune genes, but not the expression of key virulence genes in V. cholerae. Moreover, WCESP consumption did not distinctly decrease the intestinal colonization of V. cholerae in C. elegans. Taken together, our findings unveil the immune-enhancing activity of cranberry and the molecular mechanisms by which WCESP promotes *C. elegans* innate immunity against V. cholerae infection.

## Materials and Methods

### Strains and growth conditions

The bacterial strains used in this experiment were *E. coli* OP50, *V. cholerae* wild type C6706 (O1 El Tor isolated from Peru) [37], JZV133 (a *V. cholerae* strain expressing green fluorescence protein (GFP)) [38], *Staphylococcus aureus* (*S. aureus*, ATCC^#^25923), *Pseudomonas aeruginosa* (*P. aeruginosa*, ATCC^#^27853), *Enterococcus faecalis* (*E. faecalis*, ATCC^#^47077), *Salmonella typhimurium* (*S. typhimurium*, ATCC^#^14028), and *E. coli O157:H7* (ATCC^#^700927). The *E. coli*, *V. cholerae*, *P. aeruginosa*, and *S. typhimurium* strains were cultured in Luria-Bertani (LB) medium. The *S. aureus* and *E. faecalis* strains were cultured in brain-heart infusion (BHI) medium.

All *C. elegans* strains were maintained at 20°C on nematode growth medium (NGM) seeded with *E. coli* OP50 feeding strain. 100 µl of OP50 was dropped on the center of 60 mm NGM plates, which were allowed to dry overnight before the culture assays were carried out. Strains used in this study were: N2 Bristol (wild type), *sek-1(ag1)*, *pmk-1(km25)*, *daf-2 (e1370)*, *age-1(hx546), daf-16* (*mgDf50*), *hsf-1(sy441)*, *fmo-2(ok2147)*, *dod-22(ok1918)*, and CL2070(*hsp-16.2*::GFP). All the strains were obtained from the Caenorhabditis Genetics Center (CGC), University of Minnesota, USA.

### Preparation of cranberry extract

The cranberry extract used in this study was obtained from *Naturex-DBS, LLC* (Sagamore, MA, USA) and was described in previously published studies [18], [21]. Briefly, the water-soluble fractions of cranberry were spray dried, and the quality and integrity of the WCESP was standardized to 4.0% PACs. A stock solution of WCESP was freshly prepared by dissolving the powder in distilled water to a concentration of 10 mg/ml immediately before use and then the appropriate dilutions were overlaid onto the NGM plates.

### *C. elegans* killing assay

The *C. elegans* strains were routinely maintained at 20°C on nematode growth medium (NGM) agar plates seeded with *E. coli* OP50 feeding strain. Synchronized worm populations were acquired by allowing 10–15 hermaphrodites to lay eggs for 4 hours at 20°C, and then the parents were removed. *V. cholerae* was grown in freshly prepared LB medium overnight with shaking at 37°C. The overnight culture was then 10-fold concentrated and dropped (20 µl) on the center of 35 mm NGM plates (containing 50 µg/ml FUDR to prevent the growth of worm progeny) [39]–[42], which were allowed to dry for 2 hours at room temperature before killing assays were carried out. The control plates were prepared the same way by dropping 50 µl of overnight OP50 culture. For each killing assay, 25–30 synchronized worms at L4/young adult stage were transferred onto each plate and their survival was monitored thereafter. All killing assays were carried out at 25°C in triplicate and a minimum of three independent trials were performed for all strains or conditions. Day 0 was defined as the day when L4/young adult worms were transferred onto the killing assay plates. SPSS software (IBM SPSS Statistics) was used to carry out all the statistical analysis. Kaplan-Meier lifespan analysis was carried out and *p*-values were calculated using the log-rank test. *p*-value <0.05 was accepted as statistically significant.

### Gene expression analysis by quantitative real-time PCR (qRT-PCR)

All RNA samples were prepared using RNAzol RT reagent (Molecular Research Center, INC.) and stored at −80°C. Complementary DNA was synthesized using the Invitrogen Superscript first strand synthesis system for RT-PCR (Invitrogen).

For *C. elegans* immune response genes, the L1 stage nematodes were grown on NGM plates supplemented with or without 2 mg/ml WCESP at 25°C, until they reached the young adult stage. The worms were collected with M9 buffer. Transcription levels of *act-1* were used as internal controls to normalize the expression levels of target transcripts.

For *V. cholerae* virulence genes, the wild type C6706 strain was grown overnight at 37°C in LB medium supplemented with or without 2 mg/ml WCESP. The bacteria were collected and washed with PBS buffer. 16S rRNA was used as the endogenous control to normalize the expression levels of target transcripts [43].

qPCR was performed using SensiFAST SYBR No-Rox Kit (Bioline) and the CFX96 real-time PCR detection system according to the manufacturer's suggested protocol (Bio-Rad). The qPCR conditions were: 95°C for 3 minutes, followed by 40 cycles of 10 s at 95°C and 30 s at 60°C. Relative fold-changes for transcripts were calculated using the comparative CT (2^−ΔΔCT^) method [44]. Cycle thresholds of amplification were determined by Light Cycler software (Bio-Rad). Each qPCR experiment was repeated three times using independent RNA preparations. The data were pooled and analyzed using unpaired Student's t-test, and *p*<0.05 was accepted as statistically significant. The qPCR primers used in this study are listed in Table 1.

**Table 1:** Oligonucleotides used in quantitative real-time PCR.

*V. cholerae* genes		
***16s rRNA***	forward	5′-GGAAACGATGGCTAATAC CG-3′
	reverse	5 ′-GCCCTTACCTCACCAACTAG-3′
***luxO***	forward	5 ′-GCGAAAGTGGTACAGGTAAAG-3′
	reverse	5 ′-CCCTTTGACGTGACCAAAC-3′
***hapR***	forward	5 ′-CGATTGTCACTGGCTCAAAG-3′
	reverse	5 ′-GCAGTTGGTTAGTTCGGTTG-3′
***prtV***	forward	5 ′-GCTTTCAGTTCATGGGCTAAG-3′
	reverse	5 ′-GCGTGAGTTATCTGTGGTTTG-3′
***hlyA***	forward	5 ′-CCTGTAACCAAAACCTG-3′
	reverse	5 ′-CTCGCATACAAACCAAG-3′
***toxR***	forward	5 ′-GCCGATAGAAGTCATTG-3′
	reverse	5 ′-GGCATCGTTAGGGTTAG-3′
***toxT***	forward	5 ′-GGTGAATTCAAAATAAAACAGATTGC-3′
	reverse	5 ′-TCACTTGGTGCTACATTCATG-3′
***ctxA***	forward	5 ′-GTTTCTGCTTTAGGTGG-3′
	reverse	5 ′-CTCTGTAGCCCCTATTAC-3′
***tcpA***	forward	5 ′-GCTAATGGTTTGGTCAG-3′
	reverse	5 ′-CGACTGTAATTGCGAATG-3′

*C. elegans* genes		
***act-1***	forward	5 ′-CCAGGAATTGCTGATCGTATGCAGAA-3′
	reverse	5 ′-TGGAGAGGGAAGCGAGGATAGA-3′
***clec-46***	forward	5 ′-CTTCCTCGGTTCTTGCACTT-3′
	reverse	5 ′-GCGGTTTCCAACAAAAACAC-3′
***clec-71***	forward	5 ′-TTGGCTGTTGTAGGCAATCA-3′
	reverse	5 ′-TCACTGGGAATCCGTTATCC-3′
***clec-174***	forward	5 ′-CTACTTGGCAGTCTTCGCTC-3′
	reverse	5 ′-CCCATTCCAAGAGACCAGAAG-3′
***col-41***	forward	5 ′-CACCAGGAACTCCAGGAAAC-3′
	reverse	5 ′-GTGGGGTTCTGTCGTCTTGT-3′
***col-54***	forward	5 ′-ATGGAGATGTTGGAGGTGATG-3′
	reverse	5 ′-TGCTGGACATGAACAATAGGC-3′
***dct-5***	forward	5 ′-GCTGCAAAATGTGGAAATGA-3′
	reverse	5 ′-AAGTTTTGGGCACAGTCCAG-3′
***dod-22***	forward	5 ′-TTGTTGGTCCCAAGTTCACA-3′
	reverse	5 ′-AAGAACTTCGGCTGCTTCAG-3′
***fmo-2***	forward	5 ′-TGCTGTCATAGGAGCTGGTG-3′
	reverse	5 ′-CATCTGACGCCTCAAAACAA-3′
***pqn-5***	forward	5 ′-GCTCAGCCACAACAAACTCA-3′
	reverse	5 ′-CTGGCACTGTTGCTGACATT-3′
***B0024.4***	forward	5 ′-CAACAACATTGAGCGCAGAG-3′
	reverse	5 ′-TGTGTAGTCGTCTGTTGGAACC-3′
***C23G10.1***	forward	5 ′-CCATCCACTCTTGGTTGCTT-3′
	reverse	5 ′-TCACGTGCTCCTTTTTCCTT-3′
***hsp-16.2***	forward	5 ′-ATGATGAGCGTTCCAGTGATGGCTGACG-3′
	reverse	5 ′-TTAATGCATTTTTCTTGCTTCAATGTGAAGAATTCC-3′

### Intestinal colonization assay

GFP-expressing *V. cholerae* JZV133 was cultured overnight at 37°C and then seeded on NGM agar plates incorporated with and without 2 mg/ml WCESP, respectively. 10∼15 gravid worms were transferred to each plate for egg laying at 20°C, and then the parents were removed after 4 hours. Eggs were allowed to hatch and develop to adulthood on the same plates. After feeding for 3 days at 20°C, worms were picked and transferred onto LB gentamicin (50 µg/ml) plates to dwell for one hour.

To determine the fluorescent signal of bacteria in *C. elegans* intestine, worms pre-treated with or without WCESP from above LB gentamicin (50 µg/ml) plates were placed on a 2% agarose pad in a 5 µl drop of M9 buffer with 25 mM sodium azide as an anesthetic. When the worms stopped moving, a fluorescence microscope (Nikon AZ 100, Nikon, Japan) was used to examine the GFP signal.

To enumerate the bacteria in the gut, 10 worms pre-treated with or without WCESP which had spent one hour on a LB gentamicin plate were collected into a 1.5 ml Eppendorf tube and washed twice with 500 µl of sterile M9 buffer for 5 minutes with gentle rotation. Thereafter, the worms were settled and then re-suspended in 250 µl fresh M9 buffer. Silicon carbide particles (400 mg, 1.0 mm; Direct Source, Flower Mound, TX, USA) were added to disrupt the worms as previously described [45]. The worm lysate was then serially diluted and spread onto LB agar plates. The plates were incubated overnight at 37°C and the bacterial colonies were counted the next day. This assay was carried out in three independent trials. The data were pooled and analyzed using unpaired Student's t-test. *p*-value <0.05 was accepted as statistically significant.

### Western Blotting and Antibodies

Whole worm extracts were electrophoresed on a 10% SDS-PAGE gel and then transferred to a Nitrocellulose membrane using a Bio-Rad semi-dry transfer apparatus according to the manufacturer's protocols. After 30 min pre-incubation with 5% nonfat milk in TBST (10 mM Tris, pH 8.0, 150 mM NaCl, 0.5% Tween 20) at room temperature, the membrane was transferred and incubated with primary antibodies in 5% nonfat milk TBST at 4°C for 12 h. Thereafter, membrane was washed three times for 10 min and incubated with horseradish peroxidase (HRP)-conjugated secondary antibodies at room temperature. After 1.5 hours, membrane was washed with TBST three times and developed with the ECL system (Thermo Scientific) according to the manufacturer's protocols. Anti-phospho-p38 MAPK monoclonal antibody 28B10 (1∶500) was purchased from Cell Signaling. Anti-Actin monoclonal antibody MAB1501 (1∶5,000) was purchased from EMD Millipore. The goat anti-mouse IgG antibody (H&L) [HRP] (1∶10,000) was purchased from GenScript.

## Results

### WCESP supplementation prolongs *C. elegans* survival time under *Vibrio cholerae* infection

**Figure 1:**
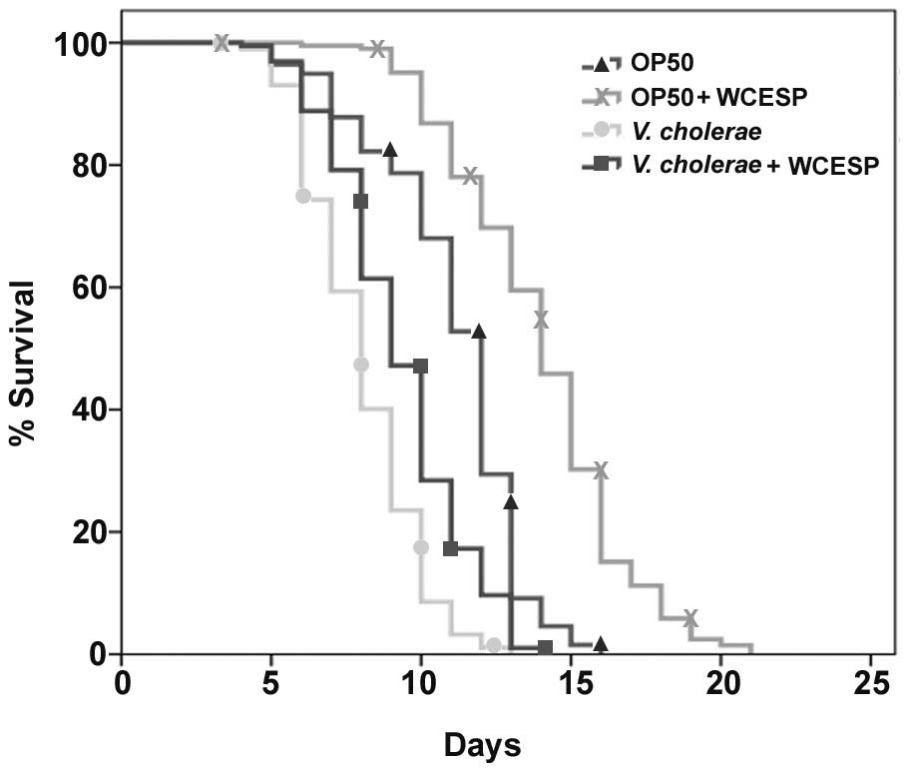
Supplementation of WCESP prolongs survival time of *C. elegans* under *V. cholerae* infection. Wild-type N2 worms were supplemented with 2 mg/ml WCESP. Each lifespan experiment was repeated in three independent trials with similar results. Quantitative data and statistical analyses for the representative experiments are included in Table 2.

**Figure 2:**
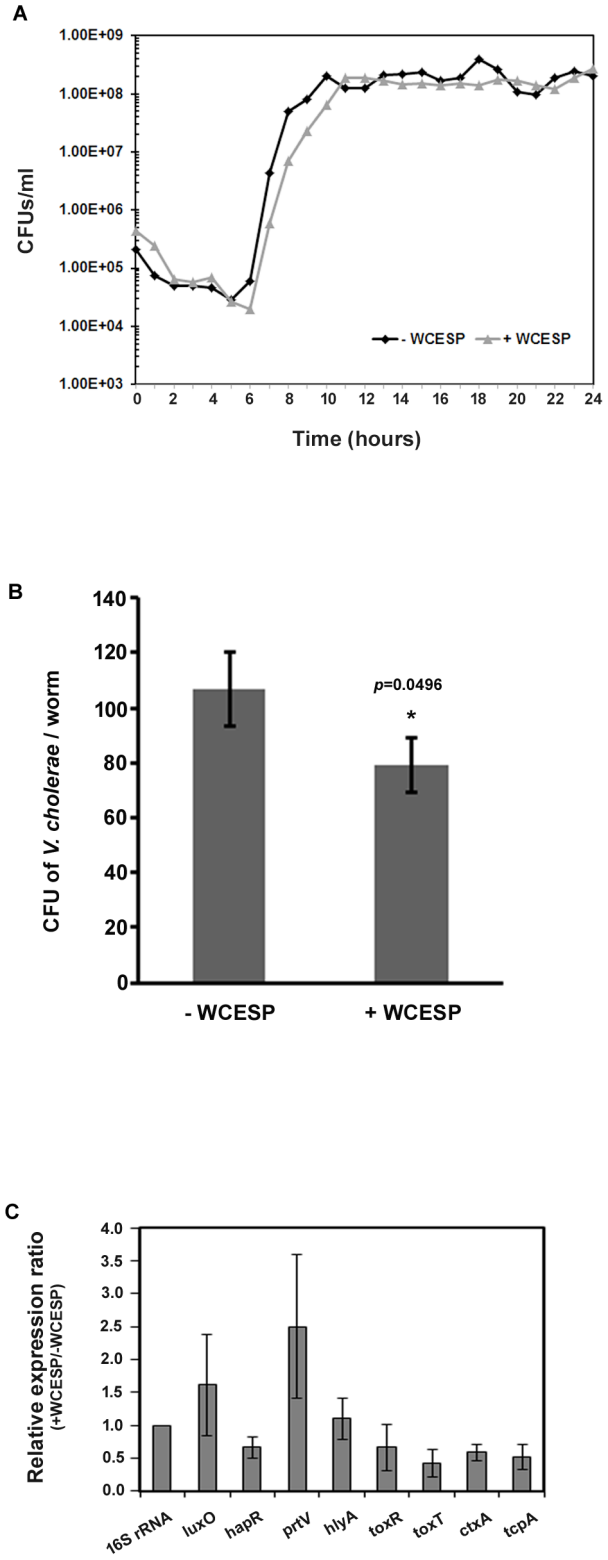
WCESP's effects on *V. cholerae*. (A) Growth of *V. cholerae* C 6706 in LB medium with and without 2 mg/ml WCESP. The experiment was repeated in three independent trials with similar results. Data shown here are representatives from one experiment. (B) WCESP slightly reduces colonization within *C. elegans* intestine. Data presented are averages from three independent trials, and error bars are standard error of the mean. *P* value was calculated using Student's *t*-test. *, *p*<0.05 when compared to the corresponding control. (C) qRT-PCR analysis of major *V. cholerae* virulence-related genes in response to 2 mg/ml WCESP (normalized to 16S rRNA). Results are the average of three independent experiments, and error bars are standard error of the mean.

Previous studies from our lab reported that 2 mg/ml of WCESP could promote worms' lifespan and thermotolerance without interfering with their physiological behaviors [18]. We hence adopted this concentration to examine whether WCESP supplementation may protect hosts from pathogenic infection by carrying out *V. cholerae* killing assays in *C. elegans*. Specifically, wild-type *C. elegans* N2 worms were supplemented with WCESP on NGM plates from the early L1 stage until L4/young adult. Thereafter, the L4/young adult wild type N2 worms were transferred and exposed to *V. cholerae* C6706 infection in the presence of WCESP. The survival of these worms was monitored every 24 hours until the last worm died. Worms cultured without WCESP supplementation served as the control. It was found that WCESP treatment resulted in significantly prolonged survival time (*p*<0.001) than that of the control, suggesting the protective effects of WCESP on *V. cholerae* infection (Fig. 1 and Table 2). In detail, WCESP consumption extended worms' mean survival time/lifespan from 7.99 days to 9.28 days when exposed to the lethal infection of *V. cholerae*. Considering the anti-microbial activities of cranberry, we wondered whether this prolonged survival time by WCESP was simply due to the killing of *V. cholerae in vitro*. To rule out this possibility, we cultured *V. cholerae* in LB medium in the presence of 2 mg/ml WCESP and monitored the growth of bacteria. Compared to the control without WCESP, we found that WCESP at 2 mg/ml did not inhibit the growth of *V. cholerae* at any of the bacterial growth phases (Fig. 2A). Therefore, our findings indicate that WCESP may possess the activities of either enhancing host immunity, inhibiting the pathogenicity of *V. cholerae*, or both.

**Table 2:** Survival of *C. elegans* N2 and various mutation strains at 25°C.

Strain	Bacterial food	Mean ± SE (Day)	Median (Day)	# of worms	*p*-value
N2^a^	*E. coli* OP50	11.08±0.25	12.0	101	
N2+WCESP^a^	*E. coli* OP50	13.98±0.28	14.0	99	<0.001
N2^a^	*V. cholerae* wt	8.06±0.20	8.0	89	
N2+WCESP^a^	*V. cholerae* wt	9.31±0.22	9.0	94	<0.001
N2^b^	*V. cholerae* wt	7.99±0.19	8.0	98	
N2+WCESP^b^	*V. cholerae* wt	9.28±0.22	9.0	103	<0.001
*pmk-1(km25)* b	*V. cholerae* wt	6.85±0.16	7.0	88	
*pmk-1(km25)*+WCESP^b^	*V. cholerae* wt	7.05±0.17	7.0	86	0.349
N2^c^	*V. cholerae* wt	8.06±0.20	8.0	89	
N2+WCESP^c^	*V. cholerae* wt	9.31±0.22	9.0	94	<0.001
*sek-1(ag1)* c	*V. cholerae* wt	6.22±0.14	6.0	93	
*sek-1(ag1)*+WCESP^c^	*V. cholerae* wt	6.40±0.16	6.0	88	0.367
N2^d^	*V. cholerae* wt	8.06±0.20	8.0	89	
N2+WCESP^d^	*V. cholerae* wt	9.31±0.22	9.0	94	<0.001
*daf-16(mgDf50)* d	*V. cholerae* wt	6.83±0.15	6.0	101	
*daf-16(mgDf50)*+WCESP^d^	*V. cholerae* wt	7.90±0.19	8.0	81	<0.001
N2^e^	*V. cholerae* wt	7.99±0.19	14.0	98	
N2+WCESP^e^	*V. cholerae* wt	9.28±0.22	15.0	103	<0.001
*hsf-1(sy441)* e	*V. cholerae* wt	6.14±0.13	6.0	98	
*hsf-1(sy441)*+WCESP^e^	*V. cholerae* wt	6.45±0.16	6.0	75	0.126
N2^f^	*V. cholerae* wt	7.34±0.21	7.0	79	
N2+WCESP^f^	*V. cholerae* wt	8.67±0.26	9.0	76	<0.001
*daf-2(e1370)* f	*V. cholerae* wt	10.94±0.26	11.0	80	
*daf-2(e1370)*+WCESP^f^	*V. cholerae* wt	11.17±0.25	11.0	82	0.386
N2^g^	*V. cholerae* wt	7.34±0.21	7.0	79	
N2+WCESP^g^	*V. cholerae* wt	8.67±0.26	9.0	76	<0.001
*age-1(hx546)* g	*V. cholerae* wt	11.41±0.24	12.0	81	
*age-1(hx546)*+WCESP^g^	*V. cholerae* wt	11.70±0.25	12.0	82	0.362

The lifespan experiments were repeated at least three times with similar results, and the data for representative experiments are shown. The lifespan data were analyzed using the log-rank test and *p*-values for each individual experiment are shown.

a Results presented in Figure 1.

b Results presented in Figure 4A.

c Results presented in Figure 4B.

d Results presented in Figure 5A.

e Results presented in Figure 5B.

f Results presented in Figure 5C.

g Results presented in Figure 5D.

### Consumption of WCESP slightly reduces *V. cholerae* colonization within *C. elegans* intestine

A number of studies have revealed that the bacterial colonization in *C. elegans* intestine plays a vital role in the process of pathogenic infection [24], [46], [47]. It is of interest to examine whether WCESP administration protects *C. elegans* from *V. cholerae* infection by attenuating the colonization of live *V. cholerae* cells in *C. elegans* intestine. To address this, a GFP-expressing *V. cholerae* strain, JZV133, was used to observe the presence of live *V. cholerae* cells inside *C. elegans* gut [38]. Briefly, GFP bacteria were fed to synchronized worms treated with WCESP, and observed via fluorescence microscopy. Non- WCESP treated worms fed with GFP bacteria, served as controls. It was found that the GFP fluorescing in the gut of worms supplemented with WCESP was similar to that of the control, indicating that supplementation of WCESP might not interfere with the bacterial colonization (data not shown). Considering that the appearance of fluorescence in the *C. elegans* intestine may not actually represent successful bacterial colonization (i.e. live bacteria) [48], we further conducted bacterial enumeration from the worm intestines. A slightly reduced number of *V. cholerae* was recovered from the WCESP treated worms than that from the control worms (79±10 vs. 107±13 CFUs/worm), and this difference is statistically significant (Fig. 2B). This suggested that the WCESP administration may impact *V. cholerae* colonization within *C. elegans* intestine, but only to a limited extent, under our experimental conditions.

### WCESP does not affect expression of major *V. cholerae* virulence genes

Previous studies elucidated that the *V. cholerae* hemolysin HlyA and the LuxO-HapR pathway-regulated extracellular protease PrtV are required for lethal infection in *C. elegans*. Both LuxO and HapR are transcriptional regulators. LuxO negatively regulates *hapR* gene expression, and HapR positively regulates *prtV* expression. Thus, a *luxO^−^* strain is hyper-toxic to the worms due to constitutive expression of HapR and the resulting overproduction of PrtV. It was also suggested that PrtV may be involved in the activation of HlyA from its protoxin form [26], [35]. We examined expression of these genes (*luxO*, *hapR*, *prtV*, and *hlyA*) that are known for worm lethality by comparing their transcriptional levels in the presence and absence of WCESP using quantitative real-time PCR (qRT-PCR). As seen in Figure 2C, there were no significant changes of these genes. We also measured expression of *ctxA*, *tcpA*, *toxT*, and *toxR* genes, which are critical for human infection but dispensable for worm killing. No apparent changes of gene expression were observed in the presence of WCESP (Fig. 2C). Taken together, our findings suggest that WCESP treatment does not affect expression of major *V. cholerae* virulence genes, at least at the transcriptional level and under our test conditions.

### WCESP modulates expression of a subset of immune response genes in *C. elegans* under non-infection condition

**Figure 3:**
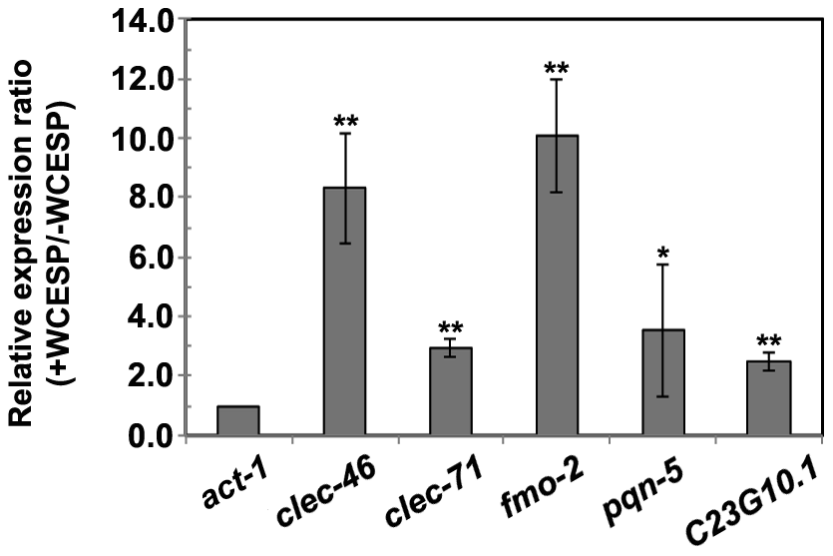
qRT-PCR analysis of *C. elegans* innate immunity genes in response to 2 mg/ml WCESP supplementation (normalized to *act-1*). Results are the average of three independent experiments, and error bars are standard error of the mean. *, *p*<0.05 when compared to the non-treated control. **, *p*<0.01 when compared to the non-treated control.

Expression of immune response genes may be induced by the WCESP, thereby enhancing worm's defense against *V. cholerae* infection. To examine this speculation, several innate immune genes (*clec-46, clec-71, clec-174, col-41, col-54, dct-5, dod-22, fmo-2, pqn-5, B0024.4*, and *C23G10.1*) that are known to respond to *V. cholerae* infection [36], were analyzed by qRT-PCR under the non-infectious condition, *i.e.* without *V. cholerae* exposure (worms were fed with *E. coli* OP50, the standard laboratory food). Under the non-infectious condition, five tested immune genes (*clec-46*, *clec-71*, *fmo-2*, *pqn-5*, and C23G10.1) were consistently induced by 2- to 10-fold in the presence of WCESP (*P*<0.05) (Fig. 3), while the other genes were not changed significantly. These data indicated that food supplementation with WCESP may result in elevated innate immunity by inducing expression of a subset of host immune genes, even when the host was not challenged with *V. cholerae* infection. In addition, we further examined the survival time of two deletion mutant worms, *fmo-2(ok2147)* and *dod-22(ok1918)*, when exposed to *V. cholerae* in the presence of WCESP. It was known that WSESP supplementation did not alter the expression of *dod-22*, while *fmo-2* was up-regulated with WCESP treatment (Fig. 3C). Intriguingly, our results showed that supplementation of WCESP significantly prolonged the survival time of both *dod-22* (from 9.07 days to 10.55 days, *p*<0.001) and *fmo-2* (from 8.53 days to 10.74 days, *p*<0.001) deletion mutant worms when exposed to *V. cholerae* infection. This finding may suggest that the immune genes regulated by WCESP function cohesively to promote host immunity in response to *V. cholerae* infection. Hence, a single gene alteration in this network may not be able to dramatically alleviate immunity induced by WCESP. Taken together, our findings suggest a general health-promoting effect of WCESP in *C. elegans* under non-infection condition.

### WCESP-mediated protection against *V. cholerae* is dependent on p38 MAPK signaling pathway

**Figure 4:**
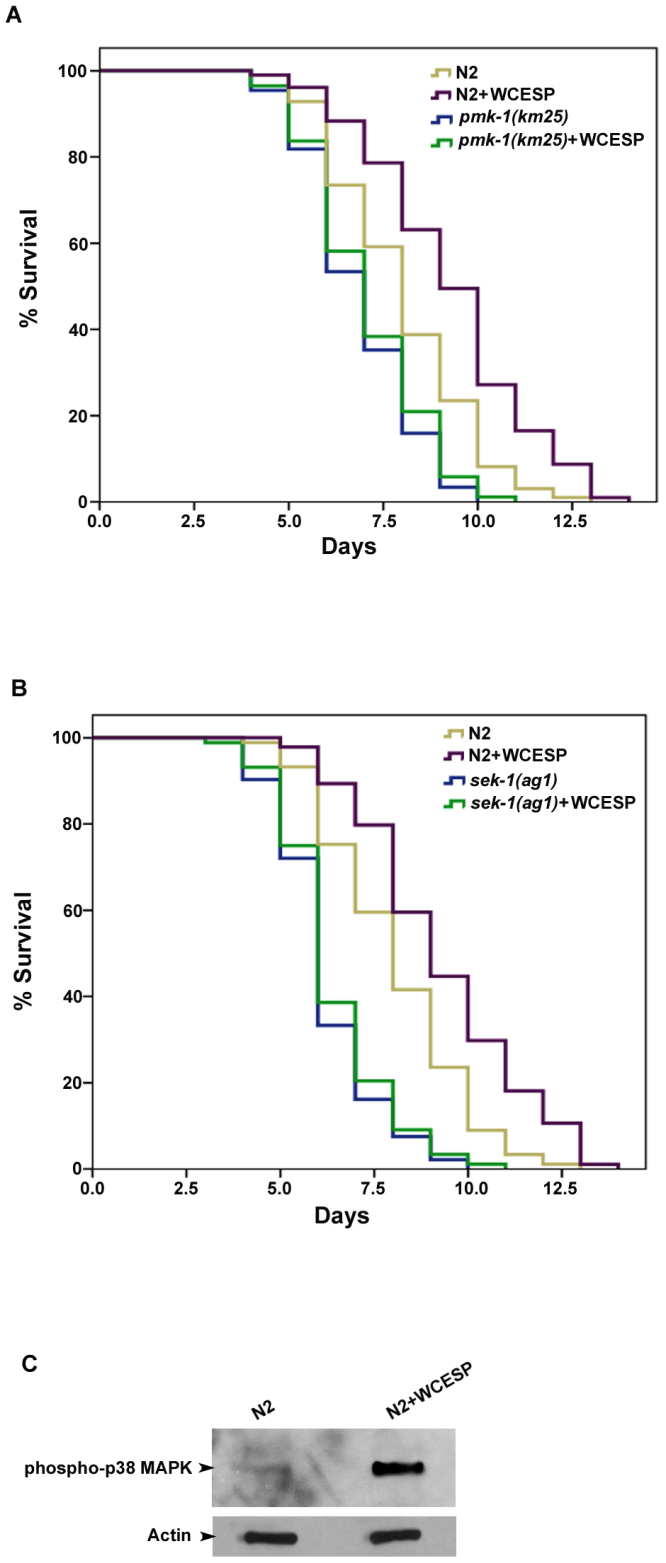
WCESP-mediated protection against *V. cholerae* is dependent on the p38 MAPK signaling pathway. (A) *pmk-1(km25)* and (B) *sek-1(ag1)* mutant worms were supplemented with 2 mg/ml WCESP. Each lifespan experiment was repeated in at least three independent trials with similar results. Quantitative data and statistical analyses for the representative experiments are included in Table 2. (C) Western blot analysis of the phosphorylated PMK-1 levels from WCESP untreated and treated worms. Actin protein was used as the loading control.

To elucidate the molecular mechanisms by which WCESP may promote innate immunity, we tested the genetic interaction of WCESP with possible immune pathways and regulators. We first conducted a genetic epistasis assay to test the relationship between WCESP and the p38 MAPK pathway, the major immunity regulatory pathway of *C. elegans* [29], [31]. We supplied null *pmk-1 (km25)* mutant worms with 2 mg/ml WCESP and found that WCESP did not extend the survival time of the *pmk-1 (km25)* mutant when exposed to *V. cholerae*, relative to the controls without WCESP supplementation (Fig. 4A, Table 2). This finding suggests that the protective effect of WCESP against *V. cholerae* infection requires functional PMK-1, the evolutionarily conserved p38 MAPK in *C. elegans*. Thereafter, we examined SEK-1, the upstream kinase of p38 MAPK. Similarly, WCESP supplementation did not extend the survival time of deletion mutant *sek-1(ag1)* worms when exposed to *V. cholerae* infection compared to the genotype matched non-supplemented controls (Fig. 4B, Table 2). These findings together suggest that WCESP acts, at least in part, through the p38 MAPK signaling pathway to protect *C. elegans* from *V. cholerae* killing.

Considering that activation of the p38 MAPK signaling pathway requires the phosphorylation of p38 MAPK, we wondered whether the phosphorylation level of PMK-1 may be elevated when worms were supplemented with WCESP. To address this, we compared the level of phosphorylated PMK-1 in wild-type worms treated with or without WCESP by western blot analysis. As shown in Fig. 4C, WCESP supplementation dramatically increased the phosphorylation of PMK-1 as compared to controls without treatment. This finding further confirmed that WCESP-mediated protection against *V. cholerae* is dependent on p38 MAPK signaling pathway.

### WCESP requires HSF-1, but not DAF-16, to promote *C. elegans* resistance to *V. cholerae* infection in an IIS dependent manner

**Figure 5:**
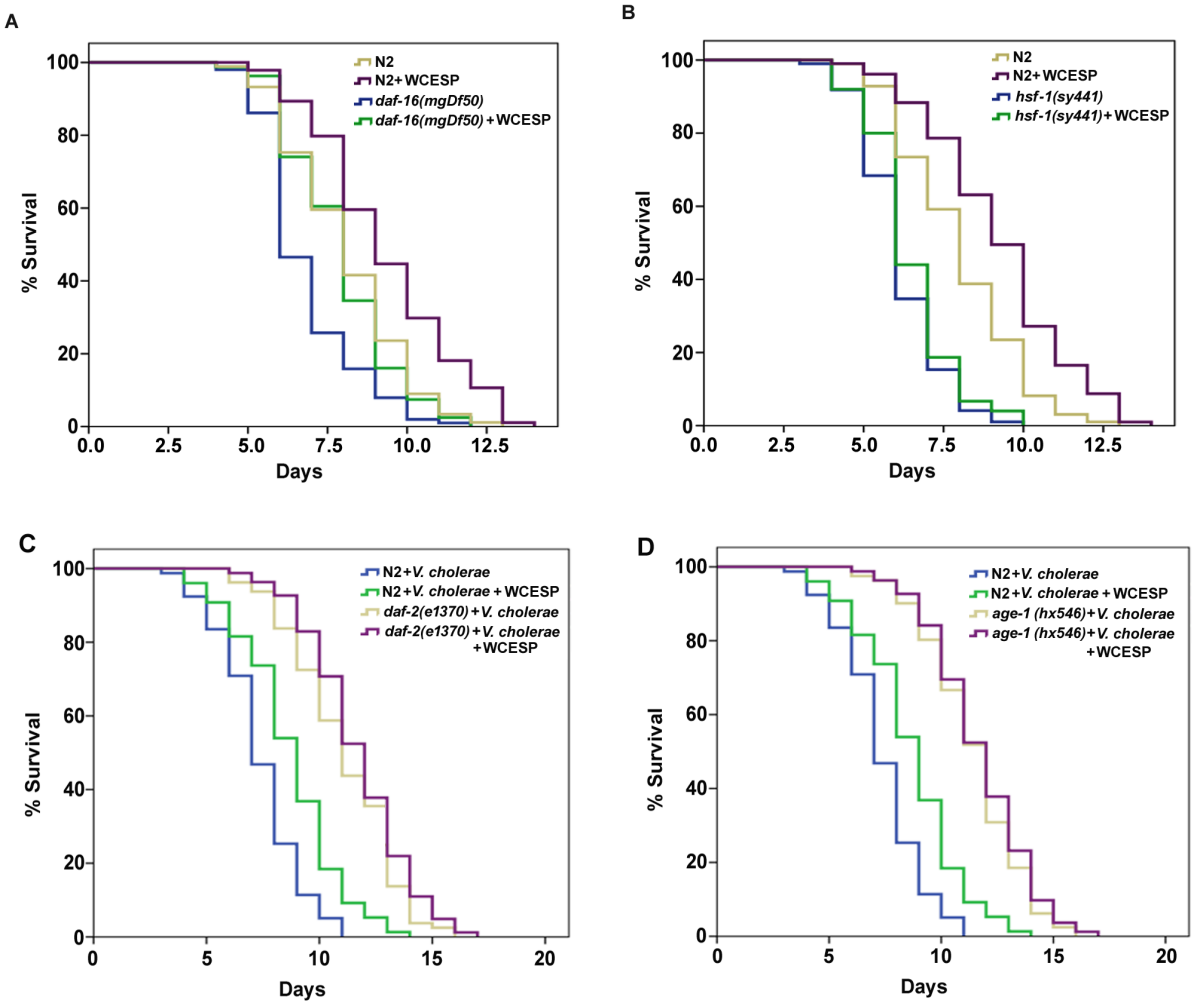
WCESP-mediated protection against *V. cholerae* requires HSF-1 and the IIS, but not Daf-16. (A) *daf-16(mgDf50)*, (B) *hsf-1(sy441)*, (C) *daf-2(e1370)*, and (D) *age-1(hx546)* mutant worms were supplemented with 2 mg/ml WCESP. Each lifespan experiment was repeated in at least three independent trials with similar results. Quantitative data and statistical analyses for the representative experiments are included in Table 2.

DAF-16 and heat-shock transcription factor (HSF) -1 are two important transcription factors that have been reported to modulate *C. elegans* immunity [30], [49]–[51]. They both generally promote worms' innate immunity and respond to infections by Gram-positive and Gram-negative bacteria. Intriguingly, supplementation of WCESP significantly extended the survival time of *daf-16* deletion mutant worms when exposed to *V. cholerae* infection (Fig. 5A, Table 2), while *hsf-1* deletion mutant worms treated with WCESP did not show prolonged survival time as compared to non-treated worms, when they were exposed to *V. cholerae* (Fig. 5B, Table 2). These results distinguished the unique role of HSF-1 in WCESP-mediated protective effect to *V. cholerae* infection. Next, we questioned whether WCESP supplementation may increase the transactivity of HSF-1 in worms. To address this, we examined the expression of *hsp-16.2*, one of the HSF-1 targets. We first visualized the green fluorescence of GFP in an *hsp-16.2::*GFP transgenic strain (CL2070) and found that these worms supplemented with WCESP showed stronger GFP signal relative to non-treated controls. Subsequently, we performed qRT-PCR to measure the mRNA level of *hsp-16.2* in wild-type N2 worms. In consistent with our GFP observation, WCESP treatment resulted in at least two-fold increase of *hsp-16.2* at transcriptional level as compared to the non-supplemented controls. These findings suggested that WCESP contribute to the protection by increasing the transactivity of HSF-1.

IIS has been reported to play a pivotal role in regulating HSF-1; we thus wondered whether WCESP might act through IIS pathway to modulate the activity of HSF-1, thereby promoting worms' immune response to *V. cholerae* infection [52]–[54]. To address this possibility, we tested *daf-2* and *age-1*, two major components of IIS pathway. Mutant worms, *daf-2(e1370)* and *age-1(hx546)*, were treated with WCESP and their survival was monitored under infection with *V. cholerae*. As compared to the genotype matched non-supplemented controls, neither *daf-2(e1370)* nor *age-1(hx546)* showed significantly prolonged survival time with WCESP supplementation (Fig. 5C & 5D, Table 2), suggesting an IIS-dependent protective effect of WCESP against *V. cholerae* infection.

## Discussion

*V. cholerae* is the causative agent of the diarrheal disease cholera [55]. Its pathogenicity in animals is mediated by numerous virulence factors, such as cholera toxin, toxin-coregulated pili, hemolysins, outer membrane adhesion factors, etc [56]. In the present study, we employed the *C. elegans* infection model to investigate the effects of WCESP on both host immunity and *V. cholerae* pathogenicity. Previously, we reported that supplementation of WCESP at a concentration of 2 mg/ml strongly promoted *C. elegans* healthspan [18], [21]. In the current study, we observed a significant protection against *V. cholerae* killing by supplying worms with the same concentration of WCESP (Fig. 1). Our studies further demonstrated that supplementation of WCESP could profoundly promote *C. elegans* innate immunity against killing by *V. cholerae* through modulating expression of host immune response genes through the p38 MAPK pathway, IIS and HSF-1. To our knowledge, this is the first study that provides molecular evidences to elucidate the immunomodulatory effects of cranberry.

The anti-microbial functions of cranberry have been extensively studied. It is known that many cranberry constituents including PACs possess activities which attenuate bacterial virulence by inhibiting their growth or adhesion ability [57]. Our results demonstrated that 2 mg/ml of WCESP treatment only slightly decreased *V. cholerae* colonization in *C. elegans* gut, and this concentration had little effect on *V. cholerae* growth and major virulence genes expression (Fig. 2). Considering that anti-microbial effects of most, if not all, cranberry constituents are dose-dependent, the concentrations of those bioactive ingredients in 2 mg/ml of WCESP may not be sufficient to affect the virulence of *V. cholerae*. We reasoned that the protective effects of 2 mg/ml WCESP on *V. cholerae* killing in *C. elegans* should be mainly reflected in the host immunity promotion.

As a bacterial predator, *C. elegans* has evolved a powerful innate immune system. A hallmark of *C. elegans* innate immunity is the activation of the p38 MAPK pathway, which is an ancestral immune pathway conserved in a variety of species [29]. Intriguingly, our epistasis assays demonstrated that WCESP required *C. elegans* PMK-1 p38 MAPK pathway to protect worms from *V. cholerae* killing (Fig. 4A, 4B, and Table 2). These findings provided genetic evidence to support the idea that WCESP supplementation may promote host innate immunity to diminish the killing of *V. cholerae* in *C. elegan*s. It is known that the p38 MAPK pathway modulates innate immunity by regulating expression of immune response genes under both pathogenic infection and noninfectious conditions [31], [58]. Given the evidence that WCESP protects *C. elegan*s from *V. cholerae* killing dependent on the p38 MAPK pathway, it is conceivable that WCESP consumption may promote host innate immunity by modulating expression of immune response genes through the p38 MAPK pathway no matter under *V. cholerae* infection or not. Troemel and her colleagues reported that C-type lectins, a group of antimicrobial polypeptides, were upregulated by active PMK-1 in *C. elegans* [31]. Sahu et al also reported that some of C-type lectins were upregulated in response to the infection of *V. cholerae* [36]. Excitingly, under noninfectious condition, we found two *C. elegans* C-type lectin genes, *clec-46* and *clec-71*, were upregulated when 2 mg/ml WCESP was supplemented (Fig. 3). Taken as a whole, our results indicate that WCESP may possess immunomodulatory effects and function through the p38 MAPK pathway to promote host innate immunity even without bacterial stimulation in *C. elegans*.

Insulin signaling and DAF-16 also play important roles in controlling *C. elegans* immunity [30], [49]. Previous studies reported that some DAF-16-regulated genes were differentially expressed in response to *V. cholerae* infection [36]. However, it was noticed that, unlike the p38 MAPK pathway, IIS and DAF-16 were thought to be a general stress response pathway rather than a specific, indispensable immunity pathway in regulating innate immunity [31]. In support of this hypothesis, our genetic results indicated that DAF-16 was dispensable for WCESP-induced immunity promotion (Fig. 5A). In addition, under the noninfectious condition, expression of *dct-5* and *dod-22*, two *V. cholerae* infection response genes which are regulated by DAF-16, were not changed with the supplementation of WCESP. Taken together, our data suggest that the immunity promoting effects of WCESP relied more on activation of the specific immunity pathway, rather than on the general stress response. Moreover, our findings further distinguished the unique roles of p38 MAPK pathway and IIS/DAF-16 in regulating *C. elegans* innate immunity.

It was shown that *C. elegans* HSF-1 is required for proper innate immunity as loss of *hsf-1* results in an increased susceptibility to pathogens [51]. However, it still remains unclear whether the HSF-1 pathway regulates a constitutive innate immunity or is only activated upon stresses caused by pathogen infection. Results from the present investigation demonstrated that HSF-1 is indispensable for WCESP-induced immunity promotion (Fig. 5B), suggesting that HSF-1 may play a vital role in maintaining a functional innate immune system in *C. elegans*. Recent studies in mammals have shown that cell stress proteins involved in the heat shock response (HSR) and endoplasmic reticulum (ER) stress response contribute to the regulation of host immunity [59]. The crosstalk of cellular stress proteins appears to manipulate the network of stress response pathways as a whole [59], [60]. Previous studies demonstrated that *V. cholerae* infection of *C. elegans* induced expression of a subset of unfolded protein response (UPR) genes, which are generally regulated by ER stress [36], [61]. In our assays, supplementation of WCESP, even without bacterial infection, elevated the expression of *C. elegans pqn-5*, an ER stress regulated response gene [36]. Considering the crosstalk between UPR and HSR, and taking into account that some UPR genes are also regulated by HSR, we hypothesized that HSF-1 may modulate *C. elegans* innate immunity in concert with UPR. Previous studies reported that increased activities of HSF-1, either by overexpression or reducing IIS, lead to an enhanced *C. elegans* innate immunity [50]. Recently, we found that WCESP acts through IIS pathway to modulate *C. elegans* lifespan [18]. These findings together implied that IIS may be important for WCESP's beneficial effects in *C. elegans*. In support of this, our results indicated that IIS (DAF-2 and AGE-1) is required for WCESP-induced immunity promotion (Fig. 5C and 5D). This finding perfectly elucidates the molecular mechanism, *i.e.* WCESP supplementation reduced the activity of IIS, thereby resulted in an elevated activity of HSF-1 and enhanced worm innate immunity against *V. cholerae*. As IIS can regulate both HSF-1 and DAF-16, the specificity of HSF-1 for WCESP's immunity promoting effects not only emphasized the importance of HSF-1 on *C. elegans* innate immunity as compared to DAF-16, but also profoundly implied the biological role of HSF-1 in regulating *C. elegans* innate immunity.

**Figure 6:**
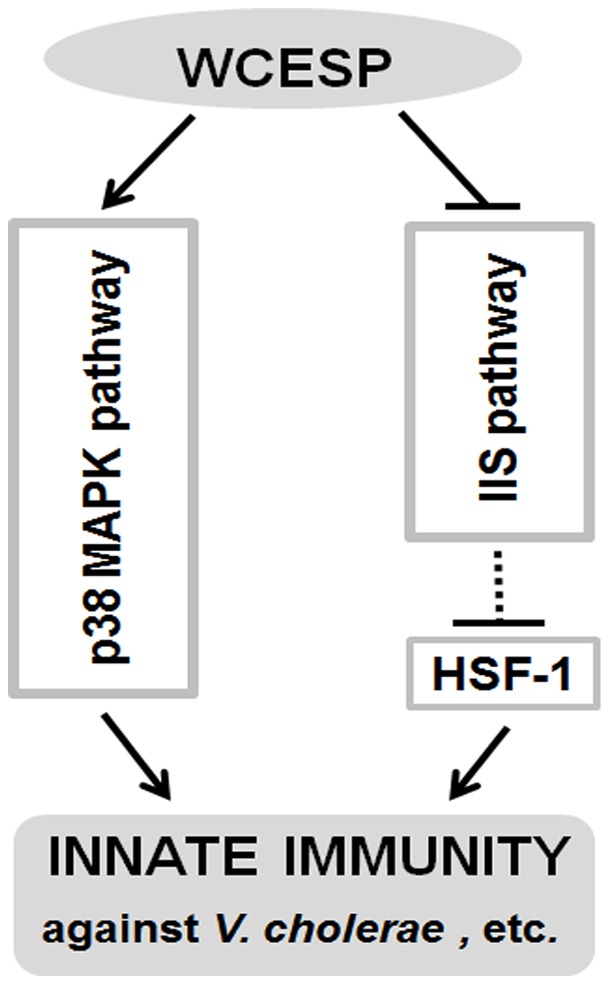
Putative mechanisms of WCESP-mediated protection against *V. cholerae* and other possible bacterial pathogens in *C. elegans* model. WCESP can stimulate the innate immunity by activating the p38 MAPK signaling pathway and by suppressing the IIS pathway which leads to an increase in HSF-1 activity.

This study systematically analyzed the genetic requirements for WCESP-mediated protective effects against *V. cholerae* infection and highlighted the innate immunity-promoting properties of WCESP. Our findings on the requirement of p38 MAPK pathway and HSF-1 not only revealed the molecular mechanisms by which WCESP promotes *C. elegans* innate immunity (Fig. 6), but also advanced our understanding of how the innate immune system is developed and maintained. Because the p38 MAPK pathway, IIS cascade, and HSF-1 are highly conserved from *C. elegans* to mammals, the WCESP-induced innate immunity promotion against *V. cholerae* may have interesting implications in higher order organisms. Interestingly, our latest studies showed that WCESP supplementation in *C. elegans* could also provide significant protection, against several other bacterial pathogens, such as *Staphylococcus aureus* and *Pseudomonas aeruginosa* (Table 3). Whether WCESP at 2 mg/ml would affect virulence of these pathogens is still under investigation, but we do know that the growth of these bacteria is not affected. As each pathogen possesses unique mode of infection, we hypothesize that the broad-spectrum pathogen protection of WCESP is mainly attributed to its immunomodulation. Certainly, further investigations are necessary to elucidate the specific mechanisms related to each pathogen protection.

**Table 3:** Survival of *C. elegans* N2 on other pathogenic bacterial strains at 25°C.

Strain	Bacterial food	Mean ± SE (Day)	Median (Day)	# of worms	*p*-value
N2	*E. coli* OP50	11.93±0.32	13.0	76	
N2+WCESP	*E. coli* OP50	13.83±0.37	15.0	78	<0.001
N2	*S. aureus*	9.57±0.37	10.0	75	
N2+WCESP	*S. aureus*	12.22±0.41	13.0	77	<0.001
N2	*P. aeruginosa*	6.47±0.18	7.0	75	
N2+WCESP	*P. aeruginosa*	8.69±0.17	9.0	84	<0.001
N2	*E. coli* OP50	11.17±0.39	11.0	76	
N2+WCESP	*E. coli* OP50	13.49±0.49	14.0	74	<0.001
N2	*E. faecalis*	9.32±0.36	9.0	72	
N2+WCESP	*E. faecalis*	10.61±0.49	11.0	74	0.001
N2	*S. typhimurium*	6.26±0.26	7.0	70	
N2+WCESP	*S. typhimurium*	7.45±0.39	8.0	74	<0.001
N2	*E. coli O157:H7*	8.67±0.41	9.0	72	
N2+WCESP	*E. coli O157:H7*	10.40±0.47	11.0	73	<0.001

The lifespan experiments were repeated at least three times with similar results, and the data for representative experiments are shown. The lifespan data were analyzed using the log-rank test and *p*-values for each individual experiment are shown.
